# Epidemiological, Clinical, and Socioenvironmental Characteristics of Cutaneous Leishmaniasis Cases in the Xakriabá Indigenous Population, Brazil

**DOI:** 10.1007/s11686-026-01312-2

**Published:** 2026-05-25

**Authors:** Dilceu Silveira Tolentino Júnior, Arlete Lisboa Gonçalves, Artur Costa Cruz, Hilana Danielle Honorato Veloso, Fabrício Xavier de Oliveira, Maelso Bispo de Sousa, Roberto Carlos de Oliveira, Eliseu Miranda de Assis

**Affiliations:** 1 Postgraduate Program in Collective Health, René Rachou Institute, Oswaldo Cruz Foundation, Belo Horizonte, Minas Gerais Brazil; 2 Regional Health Superintendency of Montes Claros, State Health Secretariat of Minas Gerais, Montes Claros, Minas Gerais Brazil; 3 São João das Missões Type 2 Base Pole, Secretariat for Indigenous Health, São João das Missões, Minas Gerais Brazil; 4 Department of Health Sciences, University of Brasília, Distrito Federal, Brasília, Brazil; 5 Academic Department, Federal Institute of Bahia, Eunápolis, Bahia Brazil

**Keywords:** Cutaneous leishmaniasis, Indigenous health, Epidemiology, Case series, Brazil, Social determinants of health

## Abstract

**Background:**

Cutaneous leishmaniasis (CL) remains a neglected tropical disease that disproportionately affects indigenous populations, where transmission is shaped by complex socioenvironmental conditions. Our objective is to describe and analyze the sociodemographic, environmental, clinical, and therapeutic characteristics of CL cases in the Xakriabá indigenous population, and to explore associations between these characteristics and clinical outcomes.

**Methods:**

An observational analytical study based on a case series was conducted using secondary data from the Brazilian Notifiable Diseases Information System (SINAN), covering the period from 2013 to 2024. Analyses were restricted to internal associations among reported cases, without inference of population-level risk. Sociodemographic, environmental, clinical, and therapeutic variables were analyzed using descriptive statistics and logistic regression models.

**Results:**

A total of 259 CL cases were identified. Most cases occurred in males (63%) and individuals aged 20–39 years (38%), with nearly all cases residing in rural areas (99%). Associations were observed between clinical outcomes and variables such as occupational exposure (OR = 2.45; 95% CI 1.38–4.33) and proximity to vegetation (OR = 2.71; 95% CI 1.49–4.92). These findings represent associations within the case population and should not be interpreted as causal effects or population risk estimates. A high proportion of missing laboratory data was identified. Spatial distribution was described without inferential analysis.

**Conclusion:**

CL in the Xakriabá population is characterized by heterogeneous distribution and associations with socioenvironmental factors within reported cases. Given the study design, results should be interpreted cautiously, without causal inference. Strengthening diagnostic capacity, improving data quality, and implementing territorially adapted public health strategies are essential to improve disease management in indigenous contexts.

## Introduction

Cutaneous leishmaniasis (CL) remains one of the most globally relevant neglected tropical diseases, with estimates ranging from 600,000 to 1 million new cases annually, disproportionately affecting populations in situations of socioeconomic vulnerability [1]. In Brazil, the disease has a wide geographic distribution and complex transmission dynamics, strongly influenced by environmental transformations, territorial occupation patterns, and persistent social inequalities [2, 3].

In recent decades, the epidemiological profile of CL has changed, with the transition from a predominantly sylvatic cycle to peridomestic and domestic patterns. This process has been associated with changes in land use, deforestation, and the expansion of human settlements in previously forested areas, intensifying the interface between humans, vectors, and reservoirs [4]. As a consequence, exposure to the vector has increased, especially in socially vulnerable populations  .

**Table 1:** Sociodemographic characteristics of CL cases in the Xakriabá indigenous population, Brazil (2013–2024)

Variable	Category	n	%
Sex	Male	162	63.0
	Female	97	37.0
Age group	≤ 19	58	22.4
	20–39	99	38.2
	40–59	65	25.1
	≥ 60	37	14.3
Residence	Rural	257	99.2
	Urban	2	0.8
Clinical form	Cutaneous	254	98.1
	Mucosal	5	1.9
Outcome	Cure	233	97.1
	Others	7	2.9

Indigenous populations constitute a group particularly exposed to CL, not only due to their proximity to natural environments, but also due to structural conditions that influence access to health services. In these contexts, the dynamics of the disease involve complex interactions between environmental factors, sociocultural practices, and territorial organization, and are not adequately explained by linear biomedical models [5, 6]. Furthermore, structural limitations, such as restricted diagnostic capacity, geographical barriers, and weaknesses in health care, can influence the time to diagnosis, clinical management, and continuity of care [7, 8].

Despite this recognized vulnerability, the literature on cutaneous leishmaniasis in indigenous populations still presents important limitations. Most available studies adopt predominantly descriptive approaches, with limited incorporation of analytical strategies that allow exploring associations between sociodemographic, environmental, and clinical characteristics. In addition, there is often a lack of clear definition of outcomes and standardization in the operationalization of variables, which restricts the comparability between studies and the robustness of interpretations [9, 10].

In the Xakriabá Indigenous Territory, located in northern Minas Gerais, cutaneous leishmaniasis constitutes a significant public health problem, in a scenario marked by intense human–environment interaction, the presence of phlebotomine vectors, and socio-environmental conditions that favor the maintenance of transmission [9]. Previous studies in the region point to the influence of ecological and cultural factors on the dynamics of the disease; however, analyses that systematically integrate epidemiological, clinical, and contextual dimensions in this territory are still scarce  .

**Table 2:** Bivariate analysis of associations between case characteristics and clinical outcomes – Xakriabá indigenous population, Brazil (2013–2024)

Variable	Category	n (%) favorable outcome*	n (%) unfavorable outcome	Crude OR (95%CI)	*p*-value
Sex	Male	150 (92.6%)	12 (7.4%)	1.87 (1.02–3.41)	0.021
	Female	83 (85.6%)	14 (14.4%)		
Occupational exposure	Yes	120 (94.5%)	7 (5.5%)	2.45 (1.30–4.60)	0.003
	No	113 (86.3%)	18 (13.7%)		
Proximity to vegetation	Yes	135 (95.1%)	7 (4.9%)	2.71 (1.45–5.07)	0.001
	No	98 (85.2%)	17 (14.8%)		
Education level	Low	139 (90.3%)	15 (9.7%)	1.62 (0.93–2.81)	0.087
	Medium/High	93 (88.6%)	12 (11.4%)		

^*^Favorable outcome defined as progress to cure. OR: odds ratio; 95% CI: 95% confidence interval

Given this context, it becomes necessary to deepen the understanding of cutaneous leishmaniasis in indigenous populations from approaches that consider the complexity of the interactions between socio-environmental determinants, clinical characteristics, and structural health conditions. This perspective can contribute to the development of surveillance and control strategies that are more sensitive to the territorial and sociocultural context. Thus, this study aimed to describe and analyze the sociodemographic, environmental, clinical, and therapeutic characteristics of cutaneous leishmaniasis cases in the Xakriabá indigenous population, as well as to explore associations between these characteristics and the observed clinical outcomes. By adopting this approach, the goal is to broaden the understanding of the epidemiological profile of the disease in this context and to support public health actions that are more appropriate to the territorial specificities (Table 3).

**Table 3:** Multivariate logistic regression model of variables uesd

Variable	Category	Adjusted OR	95% CI	*p*-value
Sex	Male	1.87	1.12–3.14	0.020
Occupational exposure	Yes	2.45	1.38–4.33	0.001
Proximity to vegetation	Yes	2.71	1.49–4.92	< 0.001

## Methods

This is an analytical observational study based on a case series, using secondary data from individuals diagnosed with cutaneous leishmaniasis (CL) belonging to the Xakriabá indigenous population, from January 1, 2013 to December 31, 2024. The study aimed to describe the epidemiological, clinical, and therapeutic profile of the cases and to explore associations between sociodemographic and environmental characteristics and clinical outcomes within the analyzed population. Considering that the sample is composed exclusively of reported cases, the analyses were restricted to internal associations, and no inferences were made about risk or patterns at the population level [11].

**Fig. 1:**
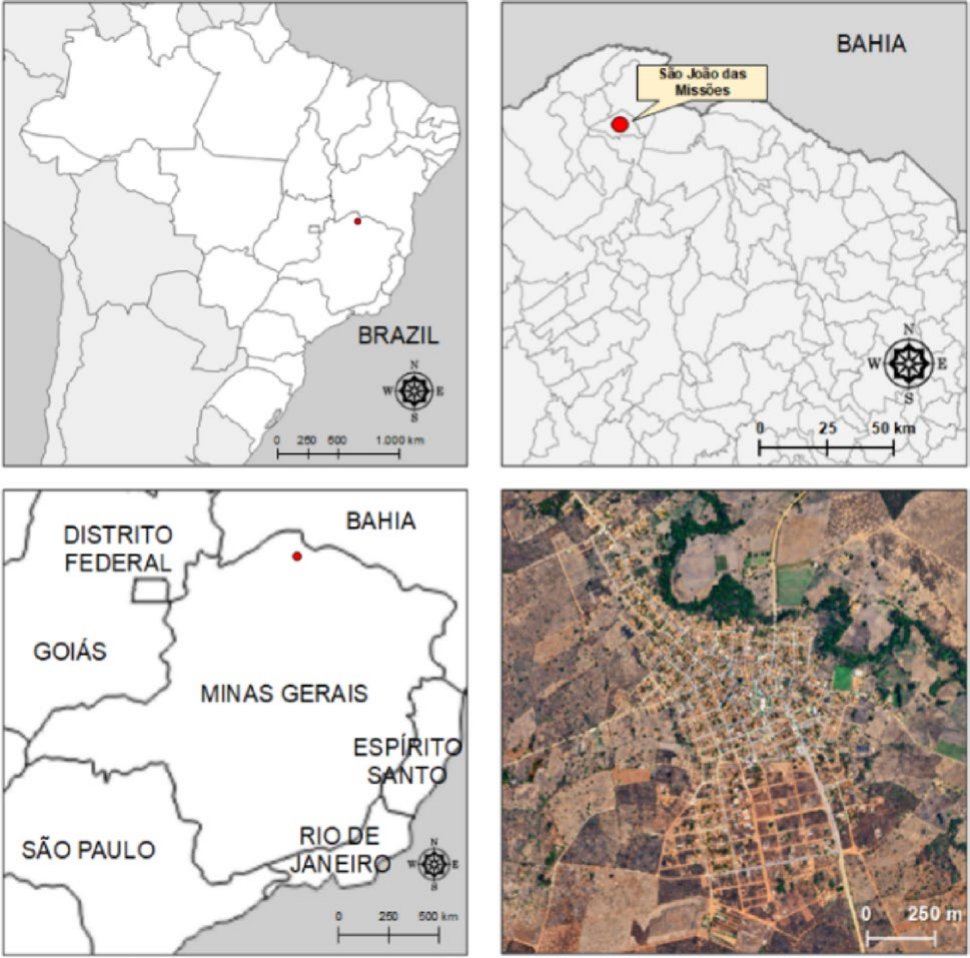
Geographic location of the Federative Republic of Brazil, state of Minas Gerais, Northern Minas Gerais region, and municipality of São João das Missões. The upper left panel indicates the location of the Federative Republic of Brazil, while the lower left panel indicates the location of the municipality of São João das Missões within the state of Minas Gerais. The upper right panel indicates the location of Northern Minas Gerais, highlighting the municipality of São João das Missões with its territorial delimitation, while the lower right panel presents a panoramic satellite aerial view of the municipality of São João das Missões

The study was conducted in the Xakriabá Indigenous Territory, located in the municipality of São João das Missões, in the north of the state of Minas Gerais, Brazil (Fig. 1). The region is characterized by the Cerrado biome, with a seasonal climate and significant interaction between the population and the natural environment, factors that influence the transmission dynamics of vector-borne diseases. The territory comprises approximately 35 villages, predominantly rural, with a strong dependence on subsistence activities such as agriculture, hunting, and gathering, which can increase exposure to phlebotomine vectors [9].

The study population included all individuals of the Xakriabá ethnic group diagnosed with cutaneous leishmaniasis reported in the Notifiable Diseases Information System (SINAN) during the study period. Cases with clinical-epidemiological and/or laboratory confirmation, residing in the indigenous territory and duly registered in the system, were considered eligible. Duplicate records, cases with inconsistencies that made it impossible to classify the main variables, and individuals without available information on the clinical outcome when necessary for analysis were excluded.

The data were obtained from SINAN, accessed through the DATASUS platform, and supplemented by technical reports from the Multidisciplinary Indigenous Health Team of the São João das Missões Base Pole. Data collection was carried out by previously trained researchers, using a standardized protocol, in order to ensure the consistency of information and minimize data extraction biases [12]. The analyses were conducted using only cases with complete data for the variables included in each model.

The study variables were organized into dependent (outcomes) and independent variables. The outcome variables considered were: clinical form of the disease (cutaneous or mucosal), therapeutic outcome (classified as cure or non-cure), and occurrence of relapse (yes/no). The independent variables included sociodemographic characteristics (age categorized as ≤ 19, 20–39, 40–59, and ≥ 60 years; sex; education; occupation), environmental and behavioral variables (area of residence classified according to registration in SINAN; proximity to vegetation areas defined based on the description of the place of residence; occupational exposure defined as activities with frequent contact with the natural environment), in addition to clinical and laboratory variables (type and number of lesions, duration of symptoms, laboratory confirmation, and HIV co-infection, when available) and therapeutic variables (medication used, adherence to treatment, and occurrence of adverse events).

The environmental and behavioral variables were defined based on information available in SINAN and standardized operational criteria to ensure greater analytical consistency. The area of residence variable (rural/urban) was classified according to registration in the notification system, following the official SINAN categorization. The variable proximity to vegetation was defined based on the declared location of the individual’s residence, being categorized as ‘‘near’’ when the dwelling was located in areas adjacent to fragments of native vegetation, forests, or agroforestry areas, and as ‘‘not near’’ when located in areas with a predominance of consolidated anthropic occupation. This classification was carried out indirectly, based on the description of the place of residence, and precise geospatial measurement was not possible.

The variable occupational exposure was defined as the performance of work or subsistence activities with frequent contact with natural environments, including agriculture, hunting, gathering, or other practices carried out in vegetation areas. Individuals who did not report such activities were classified as unexposed.

Due to the nature of secondary data, environmental variables were operationalized from available categorical information, and it was not possible to use direct quantitative measures of environmental exposure.

Statistical analysis was performed using R software, version 4.3.1 (R Foundation for Statistical Computing, Vienna, Austria). Initially, descriptive analysis was conducted using absolute and relative frequencies for categorical variables. Subsequently, associations between independent variables and outcomes were assessed using Pearson’s chi-square test or Fisher's exact test, as appropriate. Variables with a *p*-value less than 0.20 in the bivariate analysis were included in binary logistic regression models for multivariate analysis.

The results were presented as adjusted odds ratios (OR), with respective 95% confidence intervals (95% CI), adopting a significance level of 5%. Considering the study design, the measures obtained were interpreted as associations between case characteristics, without implying causal relationships or population risk estimates [11].

The spatial distribution of cases was analyzed exclusively descriptively, based on the frequency of records per village. Due to the absence of precise georeferenced data, no cluster analyses or spatial inferences were performed, and the maps were used only for illustrative purposes of the territorial distribution of cases [13].

The treatment of missing data was carried out by identifying and describing the frequency of missing information in the variables analyzed. The influence of missing data was considered in the interpretation of the results and discussed as a limitation of the study.

To minimize possible biases, strategies such as the inclusion of all eligible cases in the analyzed period were adopted, reducing selection bias; the use of a standardized data collection protocol, reducing information bias; and the use of multivariate analysis for partial control of confounding factors. The description of bias control was adjusted for greater consistency with the study design. However, it is recognized that studies based on secondary data are subject to limitations related to the quality and completeness of the information [12].

### Ethical Considerations

Because this study uses anonymized secondary data from official health information systems, the ethical principles established in Resolution No. 466/2012 of the National Health Council and in Technical Note No. 16/2020 of the Special Secretariat for Indigenous Health were respected, and review by a Research Ethics Committee was waived. All procedures were conducted in accordance with the Declaration of Helsinki.

Additionally, the clinical images presented in this study were used exclusively for scientific and educational purposes, and were duly anonymized. The images were obtained from care records and anonymized, without the possibility of identifying individuals, and were used in accordance with ethical standards for the use of secondary data in research. Free and informed consent was obtained from all participants who ceded their image rights.

## Results

259 cases of cutaneous leishmaniasis were identified and reported in the Xakriabá indigenous population between 2013 and 2024. Most cases occurred in males (63.0%) and in the 20–39 age group (38.2%). There was a predominance of residents in rural areas (99.2%). The cutaneous clinical form was identified in 98.1% of cases, while the mucosal form represented 1.9%. Regarding the therapeutic outcome, most records indicated evolution to cure (97.1%), with the remainder classified as other outcomes (failure, abandonment, or transfer), according to table 1 below. There was a significant proportion of missing data in some variables, especially education and HIV co-infection, with approximately 48% of incomplete records for the latter. 

The distribution of cases by village showed heterogeneity in the frequency of records, with a higher concentration in some specific locations. The villages with the highest number of cases included Morro Falhado (12.7%), Itapicuru (10.4%), and Imbaúba (10.0%), while others presented lower frequencies, indicating variation in the distribution of records within the territory.

Regarding laboratory data, a high proportion of tests were not performed or were not recorded: 63% of cases did not undergo parasitological examination and 81% did not undergo the Montenegro test. Among the individuals tested, 36% had a positive result for parasitological examination.

**Fig. 2:**
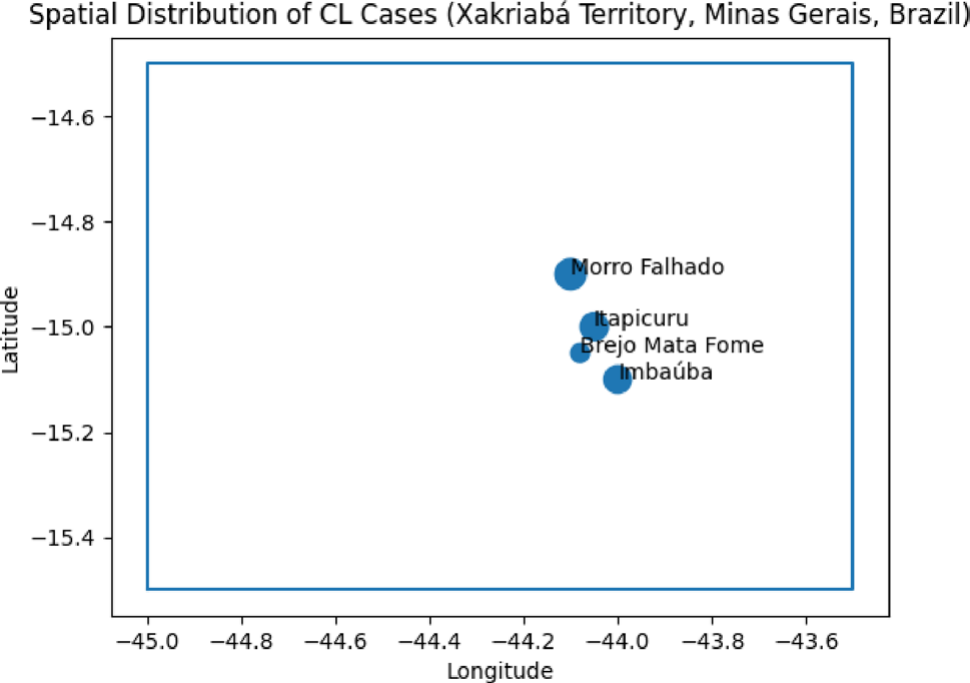
Distribution of CL cases in the Xakriabá Indigenous Territory, northern Minas Gerais, Brazil. The size of the circles is proportional to the number of cases registered per village. The map is for illustrative purposes only and was not used for spatial inference

Regarding clinical characteristics, the vast majority of individuals presented cutaneous lesions (98%), with cases of mucosal involvement being rare (2%), (Fig. 2). In cases with mucosal lesions, cutaneous scars were present in only 6 cases (2%).

The distribution of cases by village showed variation in the frequency of records within the territory, with a higher number of cases in some specific locations. The villages of Morro Falhado (12.7%), Itapicuru (10.4%), and Imbaúba (10.0%) presented a higher proportion of records in relation to the total number of cases analyzed, while other locations presented lower frequencies.

This analysis was conducted in an exclusively descriptive manner, based on the counting of cases by location, without applying statistical methods for cluster detection or spatial inference. Thus, the results do not allow for the identification of higher-risk areas or the establishment of inferential spatial patterns, and should only be interpreted as a distribution of reported cases in the territory.

A simplified cartographic base was used to represent the distribution of cases. The map is illustrative and does not accurately represent the geographic location of the villages, due to the absence of detailed georeferenced data (Fig. 2).

**Fig. 3:**
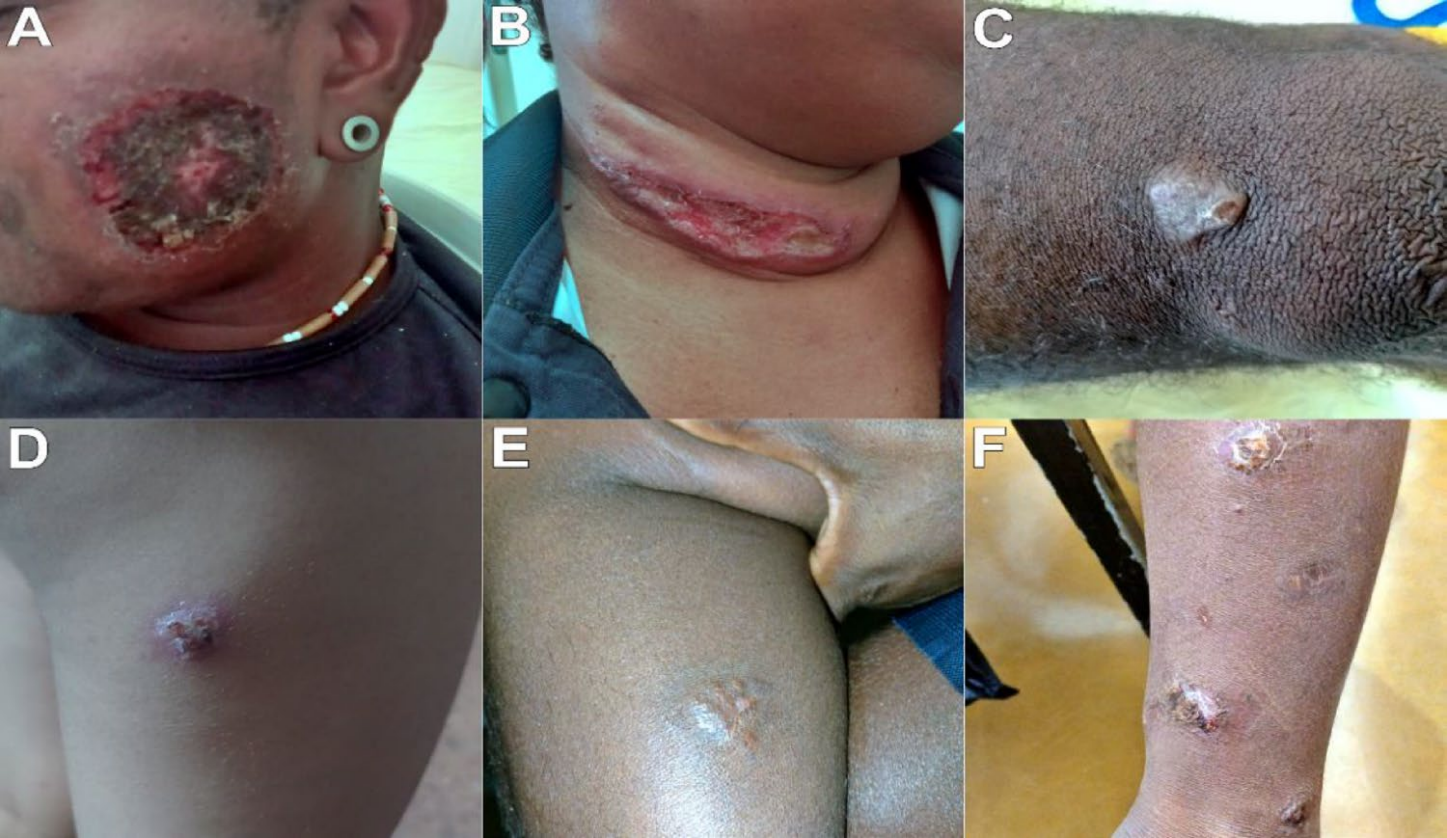
Examples of skin lesions observed in individuals of the Xakriabá indigenous population. The images were obtained from healthcare records and anonymized, without the possibility of identifying the individuals, and were used in accordance with ethical guidelines for the use of secondary data in research. Demonstration of the characteristics of the lesions: Scratch-like lesion on the left side of the face **A**, burn-like lesion **B**, keloid-like lesion above the left knee **C**, impetigo-like ulcerated lesion on the right thigh **D**, plaque-like lesion in the gastrocnemius region **E**, impetigo-like plaque-like ulcerated lesion on the right leg **F**

Skin lesions were observed in 98% of cases, while mucosal involvement was rare (2%). The characteristics of the lesions can be seen below in Fig. 3. The overall cure rate was 97%, with low rates of treatment abandonment and failure.

In the bivariate analysis, associations were observed between independent variables and the outcomes analyzed (Table 2). A statistically significant association was observed between sex and clinical outcome (*p* = 0.021), occupational exposure (*p* = 0.003), and proximity to vegetation (*p* = 0.001). The education level variable showed a borderline association (*p* = 0.087).

The bivariate analysis demonstrated statistically significant associations between the independent variables and the clinical outcomes. 

In the multivariate analysis, after adjusting for the variables included in the model, independent associations were identified between case characteristics and the outcomes analyzed (Table 3), including male sex (adjusted OR = 1.87; 95% CI 1.12–3.14; *p* = 0.020), occupational exposure (OR = 2.45; 95% CI 1.38–4.33; *p* = 0.001), and proximity to vegetation (OR = 2.71; 95% CI 1.49–4.92; *p* < 0.001). 

## Discussion

This study describes and analyzes the epidemiological, clinical, and therapeutic profile of cutaneous leishmaniasis (CL) cases in the Xakriabá indigenous population, highlighting the interaction between sociodemographic and environmental characteristics and clinical outcomes within the set of cases evaluated. The findings should be interpreted in light of the case series-based design, and it is not appropriate to infer risk or distribution of cases at the population level) [11].

In general, the results indicate a predominance of cases in young adult men residing in rural areas, a pattern consistent with previous epidemiological descriptions of CL in Brazil and in endemic contexts, in which occupational and environmental exposure plays a relevant role [3, 4]. However, these associations should be understood as observed relationships between case characteristics, without implying direct causality.

The observed association between occupational exposure, proximity to vegetation, and clinical outcomes reinforces the importance of the environmental context in the dynamics of CL. Previous studies demonstrate that changes in land use, deforestation, and expansion of inhabited areas contribute to intensifying contact between humans, vectors, and reservoirs [2, 4]. In the present study, these factors were analyzed as associated characteristics among the cases, and it was not possible to establish causal relationships or estimate the magnitude of risk.

The heterogeneous distribution of cases among the villages suggests variation in the frequency of records within the territory, which may reflect differences in environmental interaction, territorial organization, and access to health services. However, this analysis was conducted descriptively, without applying cluster detection methods or spatial inference, which limits interpretations about aggregation patterns [13]. The high proportion of missing laboratory data observed in this study highlights important limitations in diagnostic capacity in indigenous territories. Similar findings have been described in the literature, indicating that indigenous populations face persistent barriers related to access to diagnosis, health infrastructure, and care logistics [6, 7]. This limitation can influence both the classification of cases and the interpretation of results, and should be considered in the analysis of the findings.

Despite these limitations, the high proportion of cases classified as cured suggests that, when accessible, therapeutic protocols can be effective. However, this result should be interpreted with caution, considering possible limitations in data quality, such as incomplete records and potential underreporting of unfavorable outcomes. In addition, sociocultural factors, including territorial mobility and traditional care practices, can influence adherence to treatment and continuity of follow-up [14].

The results of this study should be interpreted within a broader context of socio-environmental vulnerability, in which cutaneous leishmaniasis is not limited to an isolated infectious event, but is embedded in structural dynamics involving social inequality, access to health, and environmental conditions. Recent studies have highlighted that structural determinants, such as territorial organization and social exclusion, directly influence the distribution and management of the disease [15].

When compared to other studies in Brazilian indigenous populations, the findings presented here are consistent with scenarios of persistent endemicity, although with local specificities related to the Cerrado biome and the socio-territorial characteristics of the Xakriabá [5, 16]. These differences reinforce the need for contextualized approaches, avoiding excessive generalizations among different indigenous populations.

This study has important limitations. The use of secondary data can introduce information biases resulting from incomplete or inconsistent records [12, 17, 18]. The absence of a reliable population denominator prevents the estimation of incidence and the making of inferences about risk, restricting the analysis to internal associations between cases [19]. In addition, the lack of precise georeferencing limits the performance of robust spatial analyses, and residual confounding cannot be completely excluded [20].

Despite these limitations, the study contributes by providing a structured analysis of the characteristics of cutaneous leishmaniasis (CL) cases in a specific indigenous population, incorporating multiple dimensions (epidemiological, environmental, and clinical). The findings reinforce the importance of territorially oriented public health strategies, with an emphasis on improving diagnostic capacity, enhancing information systems, and adapting surveillance actions to sociocultural specificities.

## Conclusion

This study described and analyzed the epidemiological, clinical, and therapeutic characteristics of cutaneous leishmaniasis cases in the Xakriabá indigenous population, highlighting associations between sociodemographic and environmental characteristics and clinical outcomes within the set of cases evaluated.

The findings should be interpreted considering the case series-based design, as it is not possible to establish causal relationships or infer disease risk at the population level.

A predominance of cases was observed in young adult men residing in rural areas, in addition to associations between occupational exposure, proximity to vegetation, and clinical outcomes, suggesting the relevance of the socioenvironmental context in the dynamics of the disease in this territory.

The distribution of cases showed variation among villages, analyzed descriptively, without support for spatial inference, reinforcing the need for caution in interpreting territorial patterns.

The high proportion of missing laboratory data highlights important limitations in diagnostic capacity and the quality of information systems, which may impact the classification of cases and the interpretation of results.

Despite these limitations, the results contribute to understanding the profile of cutaneous leishmaniasis in an indigenous context, indicating the importance of territorially oriented public health strategies, with strengthened surveillance, expanded diagnostic capacity, and adaptation of actions to local sociocultural specificities.

## Data Availability

The data are provided in the manuscript.
